# Evaluation of Systolic Blood Pressure, Use of Aspirin and Clopidogrel, and Stroke Recurrence in the Platelet-Oriented Inhibition in New TIA and Minor Ischemic Stroke Trial

**DOI:** 10.1001/jamanetworkopen.2021.12551

**Published:** 2021-06-04

**Authors:** Adam de Havenon, S. Claiborne Johnston, J. Donald Easton, Anthony S. Kim, Kevin N. Sheth, Maarten Lansberg, David Tirschwell, Eva Mistry, Shadi Yaghi

**Affiliations:** 1 Department of Neurology, University of Utah, Salt Lake City; 2 Dell Medical School, Austin, Texas; 3 Department of Neurology, University of California, San Francisco; 4 Department of Neurology, Yale University, New Haven, Connecticut; 5 Department of Neurology, Stanford University, Stanford, California; 6 Department of Neurology, University of Washington, Seattle; 7 Department of Neurology, Vanderbilt University, Nashville, Tennessee; 8 Department of Neurology, New York University, New York

## Abstract

**Importance:**

Elevated systolic blood pressure (SBP) after acute ischemic stroke and transient ischemic attack (TIA) is associated with future stroke risk.

**Objective:**

To explore the association of dual antiplatelet therapy (DAPT) with stroke recurrence among patients with acute ischemic stroke and TIA with or without elevated baseline SBP.

**Design, Setting, and Participants:**

This cohort study performed a post hoc subgroup analysis of the Platelet-Oriented Inhibition in New TIA and Minor Ischemic Stroke (POINT) trial, which was a multicenter trial conducted from 2010 to 2018 at 269 sites in 10 countries in North America, Europe, Australia, and New Zealand. Patients enrolled in POINT with available blood pressure and outcome data were included in this cohort. Statistical analysis was performed from November 2020 to January 2021.

**Exposures:**

Baseline SBP less than 140 mm Hg vs greater than or equal to 140 mm Hg and the interaction term of SBP (<140 mm Hg vs ≥140 mm Hg) × treatment group (aspirin vs DAPT).

**Main Outcomes and Measures:**

The primary outcome was ischemic stroke during 90 days of follow-up. The statistical analysis fit Cox proportional hazards models adjusted for patient age, race, premorbid hypertension, diabetes, and final diagnosis of the qualifying event (stroke vs TIA).

**Results:**

Among 4781 patients in the cohort, the mean (SD) age was 64.6 (13.1) years; 2142 (44.8%) were male individuals, 3487 (72.9%) were White individuals, and 266 (5.6%) had a primary outcome of ischemic stroke during follow-up. There were 946 patients (19.8%) with baseline SBP less than 140 mm Hg and 3835 (80.2%) with SBP greater than or equal to 140 mm Hg. The interaction term (SBP × treatment) was significant (*P *for interaction = .03). In the subgroup of patients with SBP less than 140 mm Hg, the hazard ratio (HR) of DAPT vs aspirin alone for ischemic stroke was 0.36 (95% CI, 0.18-0.72; *P* = .004), whereas the HR in the subgroup with SBP greater than or equal to 140 mm Hg was 0.79 (95% CI, 0.60-1.02; *P* = .08). When evaluating the outcome of ischemic stroke within 7 days of randomization, the interaction term was significant (*P *for interaction = .02), and the HR for patients with DAPT with SBP less than 140 mm Hg was 0.19 (95% CI, 0.07-0.55; *P* = .002).

**Conclusions and Relevance:**

In the POINT trial, patients with SBP less than 140 mm Hg at presentation received a greater benefit from 90 days of DAPT than those with higher baseline SBP, particularly for reduction of early ischemic stroke recurrence. Additional research is needed to replicate these findings and potentially test whether mild SBP reduction and DAPT within 12 hours of stroke onset lowers early risk of stroke recurrence.

## Key Points

**Question:**

Among patients with transient ischemic attack and stroke, what is the association between blood pressure and dual antiplatelet therapy and rates of secondary stroke within 90 days after the original ischemic event?

**Findings:**

In a post hoc analysis of 4781 patients in the POINT trial, having a baseline systolic blood pressure less than 140 mm Hg was associated with dual antiplatelet therapy significantly reducing the risk of ischemic stroke recurrence by 64% compared with aspirin alone, whereas having a baseline systolic blood pressure greater than or equal to 140 mm Hg was not associated with benefit for dual antiplatelet therapy.

**Meaning:**

Additional research is needed to replicate these findings and potentially test whether mild blood pressure reduction combined with dual antiplatelet therapy started within 12 hours of stroke onset lowers early risk of stroke recurrence.

## Teaser

This cohort study investigates whether dual antiplatelet therapy is associated with stroke recurrence among patients with or without elevated baseline systolic blood pressure in the Platelet-Oriented Inhibition in New TIA and Minor Ischemic Stroke (POINT) trial.

## Introduction

The risk of ischemic stroke in the 3 months after a minor stroke or high-risk transient ischemic attack (TIA) can exceed 10%.^1,2,3^ Patients with recurrent stroke have twice the rate of death or disability.^4,5,6^ The Clopidogrel with Aspirin in Acute Minor Stroke or Transient Ischemic Attack (CHANCE) and Platelet-Oriented Inhibition in New TIA and Minor Ischemic Stroke (POINT) trials showed that early initiation of dual antiplatelet therapy (DAPT) with aspirin and clopidogrel significantly reduced the rate of stroke events during follow-up compared with aspirin monotherapy.^7,8^ A post hoc analysis of CHANCE reported that DAPT was associated with a lower risk of stroke recurrence in patients with a baseline systolic blood pressure (SBP) greater than or equal to 140 mm Hg but did not formally test the interaction between blood pressure and treatment group, and it was limited to Asian study participants.^9^

To further evaluate the interaction between baseline blood pressure and treatment group (DAPT vs aspirin monotherapy) on secondary stroke prevention, we performed a post hoc analysis of the POINT trial. In trials of patients treated within 6 hours of ischemic stroke onset, there remains uncertainty regarding the benefit of antihypertensive therapy.^10,11,12^ The largest trial of early antihypertensive therapy in patients with ischemic stroke, the Multicenter Randomized Trial of Acute Stroke Treatment in the Ambulance With a Nitroglycerin Patch (MR ASAP), is ongoing and will enroll 1400 patients with acute ischemic stroke within 3 hours of onset and with a baseline SBP less than 140 mm Hg.^13^ Because CHANCE and POINT showed that early initiation of DAPT with suspected stroke and a SBP greater than or equal to 140 mm Hg reduced secondary stroke, we hypothesized that DAPT would be associated with a lower risk of recurrent stroke in POINT patients whose baseline SBP was less than 140 mm Hg.

## Methods

### Cohort

This cohort study of the POINT trial (NCT00991029) used a deidentified publicly available data set obtained from the National Institute of Neurologic Disorders and Stroke. Institutional review board approval and informed patient consent were not required for this deidentified data set, in accordance with 45 CFR §46. We followed the Strengthening the Reporting of Observational Studies in Epidemiology (STROBE) reporting guideline for cohort studies. POINT was a 1:1 randomized, double-blind, placebo-controlled international trial that enrolled patients at 269 sites in 10 countries with mild stroke (National Institutes of Health Stroke Scale ≤3) or high-risk TIA (ABCD^2^ [age, blood pressure, clinical features, duration of symptoms, diabetes] scale score ≥4) within 12 hours of symptom onset.^7^

Prior to enrollment, patients had to undergo a computed tomography or magnetic resonance imaging scan of the brain to rule out intracranial hemorrhage or other conditions that could explain the neurologic symptoms. Patients were ineligible if they were candidates for thrombolysis, endovascular thrombectomy, carotid endarterectomy, or anticoagulation. The 1:1 randomization was to either placebo or clopidogrel (loading dose: 600 mg followed by 75 mg daily), in addition to standard of care aspirin, and was stratified by study site. Patients were followed up for approximately 90 days (between 76 and 104 days was allowable for follow-up) after randomization. For our analysis, we included patients who had a baseline blood pressure recorded and outcome data.

### Exposures and Outcome

The exposures of interest were the treatment group (aspirin vs DAPT) and the first measured SBP (baseline SBP), which was taken in an emergency department or clinic depending on the patient’s first encounter. Given POINT’s inclusion criteria, this was a blood pressure measurement within 12 hours of symptom onset. We dichotomized SBP into less than 140 mm Hg and greater than or equal to 140 mm Hg; and for additional analysis, into 3 levels (<140 mm Hg, 140-180 mm Hg, and >180 mm Hg) that allowed examination of patients with acute hypertension (>180 mm Hg) following their stroke or TIA.^14^

The primary study outcome was ischemic stroke events during 90 days of follow-up using an intention-to-treat approach. In POINT, the primary outcome of ischemic stroke was adjudicated by 2 neurologists using data from a study outcome visit and medical record and neuroimaging review. Secondary outcomes included (1) major hemorrhage, (2) major adverse cardiovascular events (MACE), including ischemic stroke, myocardial infarction, death from a vascular cause, or major hemorrhage, and (3) ischemic stroke within 7 days of randomization. As an exploratory analysis, we used diastolic blood pressure (DBP), dichotomized into less than 90 mm Hg vs greater than or equal to 90 mm Hg, to test for associations.

### Statistical Analysis

Patient characteristics at randomization were summarized after stratification by SBP less than 140 mm Hg vs SBP greater than or equal to 140 mm Hg. We tested for intergroup differences with the *t* test for continuous variables and the χ^2^ test for categorical variables. We fit Cox proportional hazards models to our outcomes and report unadjusted and adjusted hazard ratios. The main model was adjusted for variables chosen with backward stepwise logistic regression (*P* < .05 for inclusion), including patient age, self-reported race (Black vs other), premorbid hypertension, diabetes, and final diagnosis of the qualifying event (acute ischemic stroke vs TIA). We adjusted for Black race versus other races because of a previous analysis showing a potentially confounding association.^15^ A second model was adjusted for imbalances between patients with SBP less than 140 mm Hg vs greater than or equal to 140 mm Hg, which included patient age, premorbid hypertension, congestive heart failure, statin use at 7 days from randomization, smoking status (never vs past vs current), baseline glucose and hematocrit levels, and predominant aspirin dose during the study (0 mg vs 81 mg vs 82-100 mg vs >100 mg). We confirmed the proportional hazards assumption of the adjusted Cox model on the basis of the Schoenfeld residuals of the fit model, using the estat phtest command in Stata.^16^

To test our hypothesis that the association between DAPT treatment and risk of stroke differed by the first measured blood pressure, we included an interaction term (SBP × treatment), which is the interaction of baseline SBP levels with the treatment group (aspirin vs DAPT). Subsequently, we stratified our Cox models by SBP level and report the hazard ratio for the DAPT treatment group in the stratifications. Kaplan-Meier failure curves were used to show events in the stratifications and the log-rank test to generate *P* values for the graphs. For the Kaplan-Meier curves we performed a secondary stratification by the diagnosis of the baseline qualifying event (TIA vs stroke) of the POINT trial. To account for differences between enrolling site, such as country, we fit a multilevel mixed-effects survival analysis model with a Weibull distribution and enrolling site as a random effect.^17^ Finally, as an exploratory analysis, we also included an interaction term between hypertension × treatment in our Cox models to determine whether a history of hypertension modified the association between DAPT treatment and risk of stroke. Throughout, 2-tailed tests were used with statistical significance defined as *P* < .05. All analysis was performed in Stata version 16.1 (StataCorp) from November 2020 to January 2021.

## Results

We included 4781 of the 4881 patients enrolled in POINT, with 95 patients excluded for missing outcome data and 5 excluded for absent baseline SBP. Of the 4781 patients included in the cohort, the mean (SD) age was 64.6 (13.1) years; 2142 (44.8%) were male individuals, and 3487 (72.9%) were White individuals. Additional baseline demographic characteristics are shown in Table 1, stratified by SBP less than 140 mm Hg vs SBP greater than or equal to 140 mm Hg. A total of 266 patients (5.6%) had the primary outcome of ischemic stroke during follow-up. There were 3835 patients (80.2%) with SBP greater than or equal to 140 mm Hg and 946 patients (19.8%) with SBP less than 140 mm Hg. Patients with SBP less than 140 mm Hg were significantly younger (mean [SD] age: 62.4 [14.1] years vs 65.1 [12.8] years; *P* < .001), less likely to have hypertension (504 patients [53.5%] vs 2809 patients [73.6%]; *P* < .001), less likely to be on a statin medication 7 days from randomization (699 patients [74.4%] vs 3050 patients [80.4%]; *P* < .001), and more likely to have congestive heart failure (38 patients [4.0%] vs 85 patients [2.2%]; *P* = .002) than those with SBP greater than or equal to 140 mm Hg (Table 1). The baseline demographic characteristics after stratification by the primary outcome of ischemic stroke during follow-up are seen in eTable 1 in the Supplement.

**Table 1.** Baseline Demographic Characteristics Stratified by First Measured Systolic Blood Pressure

Variable	Patients, No. (%)		*P* value^a^
	First measured systolic blood pressure		
	<140 mm Hg (n = 946)	≥140 mm Hg (n = 3835)	
Age, mean (SD), y	62.4 (14.1)	65.1 (12.8)	<.001
Sex			
Male	419 (44.3)	1723 (44.9)	.92
Female	527 (55.7)	2122 (55.1)	
Race			
White	703 (74.3)	2784 (72.6)	.50
Black	177 (18.7)	769 (20.1)	
Asian	31 (3.3)	111 (2.9)	
Other^b^	35 (3.7)	171 (4.5)	
Black race	177 (18.7)	769 (20.1)	.35
Hispanic ethnicity	86 (9.1)	289 (7.5)	.11
Final diagnosis of infarct (n = 4777)	354 (37.5)	1412 (36.9)	.73
Hypertension (n = 4761)	504 (53.5)	2809 (73.6)	<.001
Diabetes (n = 4772)	244 (25.9)	1067 (27.9)	.23
Atrial fibrillation (n = 4767)	9 (1.0)	40 (1.1)	.80
Coronary artery disease (n = 4765)	100 (10.6)	382 (10.0)	.57
Congestive heart failure (n = 4774)	38 (4.0)	85 (2.2)	.002
Carotid artery stenosis ≥50% (n = 4115)	48 (6.0)	252 (7.6)	.13
Statin at 7 d from randomization (n = 4736)	699 (74.4)	3050 (80.4)	<.001
Smoking status			
Never	470 (49.7)	2017 (52.6)	.06
Past	255 (27.0)	1052 (27.5)	
Current	221 (23.3)	763 (19.9)	
First measured, mean (SD)			
Systolic blood pressure	126.1 (10.4)	170.5 (23.1)	<.001
Diastolic blood pressure	76.1 (12.2)	91.1 (16.7)	<.001
Baseline, mean (SD)			
Glucose, mg/dL (n = 4778)	123.6 (54.9)	132.3 (62.2)	<.001
Hematocrit, % (n = 4780)	41.3 (4.8)	41.9 (4.7)	<.001
Clopidogrel treatment group	474 (50.1)	3835 (49.7)	.82
Compliant with study medication at day 7 (n = 4227)	805 (97.7)	3308 (97.2)	.44
Predominant aspirin dose during study (n = 4664)			
None	140 (3.7)	43 (4.7)	.04
81 mg	2357 (62.9)	610 (66.5)	
82-100 mg	348 (9.3)	67 (7.3)	
>100 mg	901 (24.1)	198 (21.6)	
Ischemic stroke during follow-up	42 (4.4)	224 (5.8)	.09

SI conversion factors: To convert glucose to mmol/L, multiply by 0.0555; to convert hematocrit to proportion of 1.0, multiply by 0.01.

^a^ *P* values calculated with the χ^2^ test for binary variables and *t* test for interval variables.

^b^ Other races included American Indian/Alaska Native, Native Hawaiian or other Pacific Islander, more than one race, other, and unknown or not reported.

The main adjusted Cox model fit to the primary outcome of ischemic stroke met the proportional hazards assumption. In that model, the interaction term between SBP (<140 mm Hg vs ≥140 mm Hg) and treatment group (aspirin vs DAPT) was significant in the unadjusted (*P *for interaction = .05) and adjusted (*P *for interaction = .03) models. The interaction term remained significant in the second adjusted model (*P *for interaction = .03) and third adjusted model (*P *for interaction = .02). In the multilevel mixed-effects approach to the main model with study site as the clustering variable, the interaction term was also significant (*P *for interaction = .03). However, the interaction between a history of hypertension and treatment arm was not significant in either the unadjusted (*P *for interaction = .88) or adjusted (*P *for interaction = .77) Cox models.

After stratification by SBP, DAPT’s adjusted hazard ratio (HR) for ischemic stroke in patients with SBP less than 140 mm Hg was 0.36 (95% CI, 0.18-0.72, *P* = .004), whereas with SBP greater than or equal to 140 mm Hg, the HR was 0.79 (95% CI, 0.60-1.02; *P* = .08). A similar association was seen for the outcomes of MACE and ischemic stroke within 7 days of randomization (Table 2) and for the models adjusted for baseline imbalances after the SBP stratification (eTable 2 in the Supplement). For patients with SBP less than 140 mm Hg, DAPT’s adjusted HR for ischemic stroke within 7 days was 0.19 (95% CI, 0.07-0.55; *P* = .002).

**Table 2.** Cox Proportional Hazards Models Stratified by First Measured SBP and the Model With the Interaction Term of SBP × Treatment

Outcome	Placebo event rate, No. (%)/ total No.	Clopidogrel event rate, No./total No. (%)	Unadjusted HR (95% CI)	*P* value	Adjusted HR (95% CI)^a^	*P* value	*P* value for interaction
SBP level, mm Hg							
Ischemic stroke							
SBP <140	30/472 (6.4)	12/474 (2.5)	0.39 (0.20-0.76)	.006	0.36 (0.18-0.72)	.004	.03
SBP ≥140	125/1929 (6.5)	99/1906 (5.2)	0.79 (0.61-1.03)	.09	0.79 (0.60-1.02)	.079	
Major hemorrhage							
SBP <140	4/472 (0.9)	5/474 (1.1)	1.24 (0.33-4.61)	.75	0.86 (0.21-3.44)	.83	.16
SBP ≥140	6/1929 (0.3)	18/1906 (0.9)	3.04 (1.21-7.65)	.02	3.05 (1.21-7.68)	.02	
Composite MACE outcome^b^							
SBP <140	33/482 (7.0)	17/474 (3.6)	0.50 (0.28-0.90)	.02	0.47 (0.26-0.86)	.01	.04
SBP ≥140	134/1929 (7.0)	123/1906 (6.5)	0.91 (0.72-1.17)	.47	0.91 (0.71-1.16)	.42	
Ischemic stroke within 7 d of randomization							
SBP <140	21/472 (4.5)	5/490 (1.1)	0.23 (0.09-0.61)	.003	0.19 (0.07-0.55)	.002	.02
SBP ≥140	87/1929 (4.5)	61/1906 (3.2)	0.71 (0.51-0.98)	.04	0.71 (0.51-0.97)	.03	

Abbreviations: HR, hazard ratio; MACE, major adverse cardiovascular events; SBP, systolic blood pressure.

^a^ Adjusted for patient age, Black race, premorbid hypertension, diabetes, and final diagnosis of the qualifying event (acute ischemic stroke vs transient ischemic attack).

^b^ MACE composite includes ischemic stroke, myocardial infarction, death from a vascular cause, or major hemorrhage.

**Figure 1.**
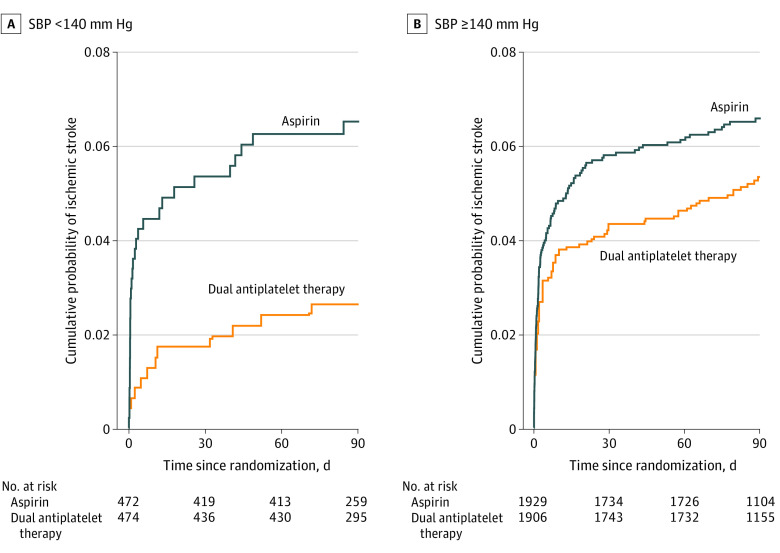
Kaplan-Meier Curves for Ischemic Stroke Events Within 90 Days, Stratified by First Measured Systolic Blood Pressure (SBP) Level With Failure Rates for Aspirin and Dual Antiplatelet Therapy

**Figure 2.**
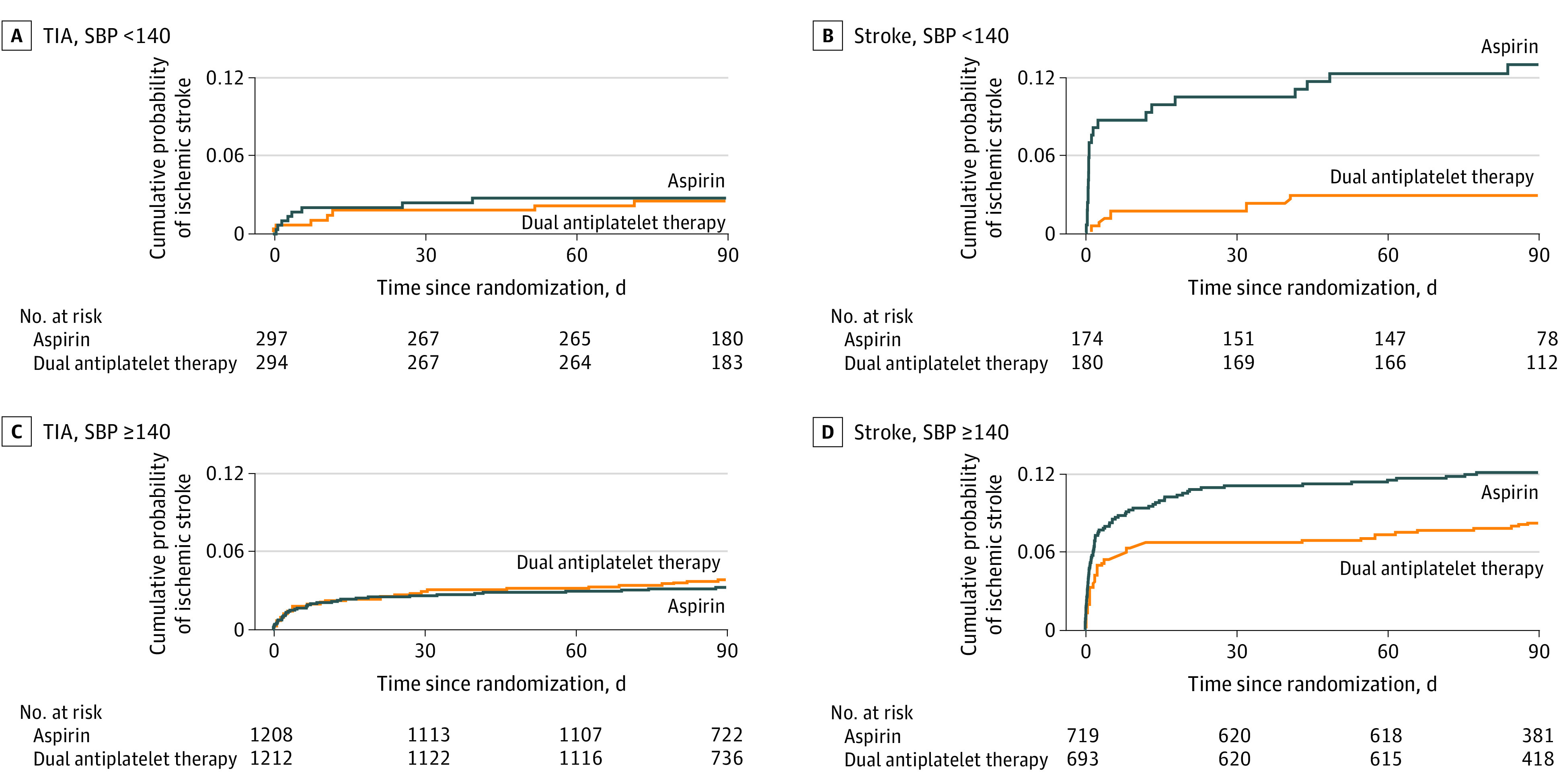
Kaplan-Meier Curves for Ischemic Stroke Events Within 90 Days, Stratified by Final Diagnosis of the Baseline Qualifying Event and First Measured Systolic Blood Pressure (SBP) With Failure Rates for Aspirin and Dual Antiplatelet Therapy TIA denotes transient ischemic attack.

The event rate of ischemic stroke in patients with SBP less than 140 mm Hg for aspirin vs DAPT was 6.4% (30 of 472) vs 2.5% (12 of 474) (*P* = .004), whereas in patients with SBP greater than or equal to 140 mm Hg it was 6.5% (125 of 1929) vs 5.2% (99 of 1906) (*P* = .09). These differences are illustrated in the Kaplan-Meier curves seen in Figure 1, which show the reduction in early stroke events seen in patients with SBP less than 140 mm Hg on DAPT and nearly identical curves for the SBP stratifications in the aspirin treatment group (Figure 1). This was even more apparent after the second stratification by the trial’s qualifying event (baseline stroke vs TIA), which revealed no separation by treatment group for TIA patients, but a reduction in early stroke events for patients with SBP less than 140 mm Hg on DAPT who had acute stroke as the qualifying event, and a less impressive separation for SBP greater than or equal to 140 mm Hg, with HRs of 0.21 (95% CI, 0.08-0.55) vs 0.65 (95% CI, 0.46-0.91), respectively (Figure 2).

Although the interaction term of SBP × treatment was not significant for the secondary outcome of major hemorrhage (*P* for interaction = .16), patients randomized to DAPT with SBP greater than or equal to 140 mm Hg had an HR for major hemorrhage of 3.05 (95% CI, 1.21-7.68; *P* = .02), whereas patients with SBP less than 140 mm Hg demonstrated no association. In the stratification by SBP less than 140 mm Hg, 140 to 180 mm Hg, and greater than 180 mm Hg (eTable 3 in the Supplement), we observed a rate of stroke in the aspirin group of 6.4% (30 of 472), 5.2% (71 of 1357), and 9.4% (54 of 572), respectively. Although patients with a SBP greater than 180 mm Hg had the highest rate of stroke during follow-up, there was not a significant association between DAPT treatment and risk of stroke (HR for ischemic stroke, 0.70; 95% CI, 0.46-1.05; *P* = .09). However, the association between DAPT and stroke risk with SBP from 140 to 180 mm Hg was also less apparent (HR for ischemic stroke, 0.84; 95% CI, 0.60-1.19; *P* = .32). Of note, the secondary outcome of major hemorrhage was not increased in patients with SBP greater than 180 mm Hg who were randomized to DAPT (eTable 3 in the Supplement).

The exploratory stratification of DBP less than 90 mm Hg vs greater than or equal to 90 mm Hg was less informative (Table 3) because the interaction terms between DBP and treatment arm were not significant, apart from the model fit to ischemic stroke within 7 days. In that model, the interaction term was significant (*P* for interaction = .02) and, after stratification, in patients with DBP less than 90 mm Hg the HR of DAPT for ischemic stroke was 0.38 (95% CI, 0.24-0.62; *P* < .001), whereas with DBP greater than or equal to 90 mm Hg, the HR was 0.83 (95% CI, 0.55-1.26; *P* = .36).

**Table 3.** Cox Proportional Hazards Models Stratified by First Measured DBP Level and the Model With the Interaction Term of DBP × Treatment

Outcome	Placebo event rate, No./total No. (%)	Clopidogrel event rate, No./total No. (%)	Unadjusted HR (95% CI)	*P* value	Adjusted HR (95% CI)^a^	*P* value	*P* value for interaction
DBP level, mm Hg							
Ischemic stroke							
DBP <90	78/1342 (5.8)	51/1326 (3.9)	0.65 (0.46-0.93)	.02	0.63 (0.44-0.90)	.01	.45
DBP ≥90	77/1059 (7.3)	60/1054 (5.7)	0.78 (0.55-1.09)	.14	0.76 (0.54-1.07)	.12	
Major hemorrhage							
DBP <90	6/1342 (0.5)	17 (1326 (1.3)	2.88 (1.13-7.30)	.03	2.62 (1.03-6.72)	.04	.51
DBP ≥90	4/1059 (0.4)	6/1054 (0.6)	1.50 (0.42-5.33)	.53	1.54 (0.43-5.47)	.51	
Composite MACE outcome^b^							
DBP <90	84/1342 (6.3)	70/1326 (5.3)	0.82 (0.60-1.13)	.22	0.79 (0.58-1.09)	.16	.87
DBP ≥90	83/1059 (7.8)	70/1054 (6.6)	0.84 (0.61-1.16)	.29	0.91 (0.60-1.14)	.24	
Ischemic stroke within 7 d of randomization							
DBP <90	58/1342 (4.3)	24/1326 (1.8)	0.41 (0.26-0.67)	<.001	0.38 (0.24-0.62)	<.001	.02
DBP ≥90	50/1059 (4.7)	42/1054 (4.0)	0.84 (0.56-1.27)	.40	0.83 (0.55-1.26)	.39	

Abbreviations: DBP, diastolic blood pressure; HR, hazard ratio; MACE, major adverse cardiovascular events.

^a^ Adjusted for patient age, Black race, premorbid hypertension, diabetes, and final diagnosis of the qualifying event (acute ischemic stroke vs transient ischemic attack).

^b^ MACE composite includes ischemic stroke, myocardial infarction, death from a vascular cause, or major hemorrhage.

## Discussion

In this post hoc cohort study of the POINT trial, we found that patients with baseline SBP less than 140 mm Hg at presentation had a more consistent association between DAPT (clopidogrel and aspirin) and lower recurrent stroke risk than patients with higher baseline SBP. The association was present for the outcome of ischemic stroke and for the composite outcome of MACE. When considering the larger effect size for ischemic stroke within 7 days, the reduction of early events on the Kaplan-Meier curves, and the association in patients with acute stroke as the qualifying event, the mechanism of DAPT in patients with SBP less than 140 mm Hg appears to be mitigation of early stroke recurrence. An analysis after stratification by baseline DBP less than 90 mm Hg vs greater than or equal to 90 mm Hg did not produce consistently significant interactions with the treatment group. The interaction between a history of hypertension and treatment group was not significant, suggesting the observed association is related to poststroke blood pressure more than underlying hypertension.

The post hoc analysis of the CHANCE trial by Xu et al^9^ reported that the association between DAPT and lower risk of recurrent stroke was only significant in patients with baseline SBP greater than or equal to 140 mm Hg.^9^ In both our analysis and that by Xu et al^9^ analysis, the direction of effect was toward an association between DAPT and lower risk of recurrent stroke, regardless of baseline SBP. Although the analysis by Xu et al^9^ did not report a formal interaction term for SBP treatment, our analysis found a consistent interaction, and stratified models showed the association between DAPT and lower risk of recurrent stroke was only significant in patients with baseline SBP less than 140 mm Hg.

There were differences in the CHANCE and POINT cohorts, which led to a 90-day stroke rate of 10% in CHANCE vs 5.5% in POINT.^7,8^ The most prominent difference is that patients in CHANCE were more likely to have intracranial atherosclerosis given the higher rates in Asian patients with stroke.^18^ Patients with intracranial atherosclerotic stenosis may require higher blood pressure in the subacute period after stroke to prevent recurrent stroke or extension of the initial stroke from hypoperfusion. However, in the analysis of CHANCE by Xu et al^9^ they were not able to fully explore the association of intracranial atherosclerosis because of limited imaging data, and they did not explore the association of the qualifying event (stroke vs TIA) or early recurrence. Additional differences between the CHANCE and POINT cohorts include a lower rate of smoking in POINT participants and higher rate of diabetes.

One possible explanation for our finding that DAPT was associated with lower risk of recurrent stroke risk in patients with baseline SBP less than 140 mm Hg is a synergistic relationship between lower blood pressure during the acute and subacute stages of stroke and the antithrombotic effects of DAPT in the week after the index event. Trials of blood pressure lowering in the days after ischemic stroke onset have consistently been neutral to negative.^10,11,19,20,21^ However, those trials do not preclude the possibility that moderate blood pressure control (target SBP less than 140 mm Hg or less than 160 mm Hg) combined with the antithrombotic properties of DAPT could lower the risk of early stroke recurrence by targeting vascular risk in tandem, both stabilizing the endothelium and reducing embolic events.^22,23,24^

Another explanation is that the lower baseline blood pressure may have been a biomarker of unmeasured confounding, although the 90-day rates of stroke in the aspirin monotherapy group were similar for SBP less than 140 mm Hg vs greater than or equal to 140 (6.4% vs 6.5%). After stratification by SBP less than 140 mm Hg vs 140 to 180 mm Hg vs greater than 180 mm Hg, we observed the familiar U-shaped association of stroke risk in the aspirin group (6.4% vs 5.2% vs 9.4%),^25,26^ suggesting that the most protective range of SBP may be within the range of 140 to 180 mm Hg. It is also possible that SBP greater than 140 mm Hg was a marker of a higher proportion of lacunar strokes. In the Secondary Prevention of Small Subcortical Strokes trial, the use of DAPT did not prevent recurrence in patients with lacunar stroke, although the median time from index stroke to randomization was 62 days, which would have prevented measuring a reduction in early events.^27^

### Limitations and Strengths

This cohort study has limitations that restrict its validity and generalizability. The first is that this is a post hoc analysis of a randomized clinical trial that was not designed to answer the proposed question, which introduces inherent bias. In the POINT trial, the stroke or TIA mechanism was not recorded, preventing analyses specific to subtype, such as exploring the proportion of lacunar stroke in patients with SBP greater than or equal to 140 mm Hg. We do not have additional blood pressure measurements during the days after stroke onset, which would be necessary to fully evaluate the observed association. We also cannot account for medications that may have been given prior to the baseline blood pressure measurement, although the exclusion of patients who were eligible for tissue plasminogen activator in POINT indicates that most patients would not have received antihypertensives, consistent with American Heart Association guidelines.^28^ Several strengths of our study warrant mention, including high-quality outcome adjudication and a diverse cohort with high medication compliance and minimal loss to follow-up.

## Conclusions

In this post hoc analysis of the POINT trial, patients with SBP less than 140 mm Hg at presentation received a greater benefit from 90 days of DAPT than those with higher baseline SBP, particularly for reduction of early ischemic stroke recurrence. The findings of this analysis contradict a prior publication on the CHANCE trial and, thus, could be due to chance. Additional research is needed to replicate our findings and potentially test whether mild SBP reduction and DAPT within 12 hours of stroke onset lowers early risk of stroke recurrence.

## Notes

Supplement. **eTable 1.** Baseline Demographic Characteristics in Patients With and Without Ischemic Stroke in the 90 Days After Randomization **eTable 2.** Cox Proportional Hazard Models Stratified by First Measured Systolic Blood Pressure (SBP) Level (<140 vs ≥140 mm Hg) **eTable 3.** The Effect of Randomization to Placebo vs Clopidogrel by First Measured Systolic Blood Pressure (SBP) Level (SBP <140 vs 140-180 vs >180 mm Hg)
