# Supplementary material for: Evaluation of Systolic Blood Pressure, Use of Aspirin and Clopidogrel, and Stroke Recurrence in the Platelet-Oriented Inhibition in New TIA and Minor Ischemic Stroke Trial
Source: JAMA Netw Open. 2021 Jun 4;4(6):e2112551. doi: 10.1001/jamanetworkopen.2021.12551 (PMC8178708; doi:10.1001/jamanetworkopen.2021.12551)
Supplement: Supplement. — eTable 1. Baseline Demographic Characteristics in Patients With and Without Ischemic Stroke in the 90 Days After Randomization eTable 2. Cox Proportional Hazard Models Stratified by First Measured Systolic Blood Pressure (SBP) Level (<140 vs ≥140 mm Hg) eTable 3. The Effect of Randomization to Placebo vs Clopidogrel by First Measured Systolic Blood Pressure (SBP) Level (SBP <140 vs 140-180 vs >180 mm Hg) [file jamanetwopen-e2112551-s001.pdf]

Supplemental Online Content

de Havenon A, Johnston SC, Easton JD, et al. Evaluation of systolic blood pressure, use of aspirin and clopidogrel, and stroke recurrence in the Platelet-Oriented Inhibition in New TIA and Minor Ischemic Stroke trial. *JAMA Netw Open.* 2021;4(6):e2112551. doi:10.1001/jamanetworkopen.2021.12551

eTable 1. Baseline Demographic Characteristics in Patients With and Without Ischemic Stroke in the 90 Days After Randomization

eTable 2. Cox Proportional Hazard Models Stratified by First Measured Systolic Blood Pressure (SBP) Level (<140 vs ≥140 mm Hg)

eTable 3. The Effect of Randomization to Placebo vs Clopidogrel by First Measured Systolic Blood Pressure (SBP) Level (SBP <140 vs 140-180 vs >180 mm Hg)

This supplemental material has been provided by the authors to give readers additional information about their work.

© 2021 de Havenon A et al. *JAMA Network Open.*

eTable 1. Baseline demographic characteristics in patients with and without ischemic stroke in the 90 days after randomization.

**Variable**	**No ischemic stroke during follow-up (n=4,515)**	**Ischemic stroke during follow-up (n=266)**	**p value***
Age	64.5±13.1	66.2±12.5	0.04
Male sex	2,022 (44.8%)	120 (45.1%)	0.92
Race<br>White<br>Black<br>Asian<br>Other	 3,314 (73.4%)<br>868 (19.2%)<br>137 (3.0%)<br>196 (4.3%)	 173 (65.0%)<br>78 (29.3%)<br>5 (1.9%)<br>10 (3.8%)	0.001
Black race	868 (19.2%)	78 (29.3%)	<0.001
Hispanic ethnicity	357 (7.9%)	18 (6.8%)	0.50
Final diagnosis of infarct (n=4,777)	1,598 (35.4%)	168 (63.4%)	<0.001
Hypertension (n=4,761)	3,101 (69.0%)	212 (80.0%)	<0.001
Diabetes (n=4,772)	1,212 (26.9%)	99 (37.2%)	<0.001
Atrial fibrillation (n=4,767)	46 (1.0%)	3 (1.1%)	0.86
Coronary artery disease (n=4,765)	456 (10.1%)	26 (9.8%)	0.87
Congestive heart failure (n=4,774)	116 (2.6%)	7 (2.6%)	0.95

© 2021 de Havenon A et al. *JAMA Network Open.*

Carotid artery stenosis ≥50% (n=4,115)	259 (6.7%)	41 (17.2%)	<0.001
Statin at 7 days from randomization (n=4,736)	3,526 (78.7%)	223 (86.8%)	0.002
Smoking status			0.53
Never	2,346 (52.0%)	141 (53.2%)	
Past	1,242 (27.5%)	65 (24.5%)	
Current	925 (20.5%)	59 (22.3%)	
First measured systolic blood pressure	161.3±27.2	169.8±32.9	<0.001
First measured diastolic blood pressure	87.9±16.8	91.0±19.7	0.004
First measured systolic blood pressure >180	1,043 (23.1%)	92 (34.6%)	<0.001
Baseline glucose (n=4,778)	129.5±60.1	149.7±70.9	<0.001
Baseline hematocrit (n=4,780)	41.8±4.8	41.5±4.6	0.35
Clopidogrel treatment arm	2,269 (50.3%)	111 (41.7%)	0.007
Compliant with study medication at day 7 (n=4,227)	3,924 (97.3%)	194 (98.0%)	0.55
Predominant aspirin dose during study (n=4,664)			<0.001
None	166 (3.8%)	17 (6.6%)	
81 mg	2,827 (64.1%)	140 (54.2%)	
82-100 mg	397 (9.0%)	18 (7.0%)	
>100 mg	1,016 (23.1%)	83 (32.2%)	

* Binary variables presented as n (%); ordinal variables as median, IQR; interval variables as mean±standard deviation. P values calculated with the chi-squared test for binary variables, the Wilcoxon rank sum test for ordinal variables, and Student's t-test for interval variables.

© 2021 de Havenon A et al. *JAMA Network Open.*

eTable 2. Cox proportional hazard models stratified by first measured systolic blood pressure (SBP) level (<140 vs. ≥140 mm Hg) showing hazard ratios adjusted for patient age, premorbid hypertension, congestive heart failure, statin use at 7 days from randomization, smoking status, baseline glucose and hematocrit, and predominant aspirin dose during the study.

**Outcome**	**SBP level**	**Adjusted hazard ratio (95% CI)**	**p value**
Ischemic stroke	SBP <140	0.32 (0.16-0.66)	0.002
	SBP ≥140	0.77 (0.59-1.01)	0.06
Major hemorrhage	SBP <140	0.81 (0.20-3.33)	0.77
	SBP ≥140	3.16 (1.25-7.99)	0.02
Composite MACE outcome*	SBP <140	0.44 (0.24-0.82)	0.009
	SBP ≥140	0.90 (0.70-1.16)	0.41
Ischemic stroke within 7 days	SBP <140	0.19 (0.07-0.56)	0.003
	SBP ≥140	0.68 (0.49-0.96)	0.03

*MACE composite includes ischemic stroke, myocardial infarction, death from a vascular cause, or major hemorrhage

© 2021 de Havenon A et al. *JAMA Network Open.*

eTable 3. The effect of randomization to placebo vs clopidogrel by first measured systolic blood pressure (SBP) level (SBP <140 vs. 140-180 vs. >180 mm Hg).

Outcome	SBP level	Placebo event rate (n, %)	Clopidogrel event rate (n, %)	Unadjusted hazard ratio (95% CI)	p value	Adjusted hazard ratio** (95% CI)	p value
Ischemic stroke	<140	30/472, 6.4%	12/474, 2.5%	0.39 (0.20-0.76)	0.006	0.36 (0.18-0.72)	0.004
	140-180	71/1357, 5.2%	61/1343, 4.5%	0.86 (0.61-1.21)	0.340	0.84 (0.60-1.19)	0.32
	>180	54/572, 9.4%	38/563, 6.8%	0.70 (0.46-1.06)	0.09	0.70 (0.46-1.05)	0.09
Major hemorrhage	<140	4/472, 0.9%	5/474, 1.1%	1.24 (0.33-4.61)	0.75	0.86 (0.21-3.44)	0.83
	140-180	3/1357, 0.2%	14/1343, 1.0%	4.74 (1.36-16.48)	0.02	4.64 (1.33-16.18)	0.02
	>180	3/572, 0.5%	4/563, 0.7%	1.34 (0.30-5.99)	0.70	1.36 (0.31-6.11)	0.69
Composite MACE outcome*	<140	33/472, 7.0%	17/474, 3.6%	0.50 (0.28-0.90)	0.02	0.47 (0.26-0.86)	0.01
	140-180	74/1357, 5.5%	81/1343, 6.0%	1.09 (0.80-1.50)	0.59	1.06 (0.78-1.46)	0.70
	>180	60/572, 10.5%	42/563, 7.5%	0.70 (0.47-1.03)	0.07	0.69 (0.46-1.02)	0.06
Ischemic stroke within 7 days	<140	21/472, 4.5%	5/474, 1.1%	0.23 (0.09-0.62)	0.003	0.19 (0.07-0.55)	0.002
	140-180	49/1357, 3.6%	36/1343, 2.7%	0.74 (0.48-1.14)	0.17	0.72 (0.47-1.11)	0.13
	>180	38/572, 6.6%	25/563, 4.4%	0.66 (0.40-1.09)	0.11	0.65 (0.39-1.08)	0.010

*MACE composite includes ischemic stroke, myocardial infarction, death from a vascular cause, or major hemorrhage
** Adjusted for patient age, Black race, premorbid hypertension, diabetes, and final diagnosis of the qualifying event (acute ischemic stroke vs. TIA)

© 2021 de Havenon A et al. *JAMA Network Open.*
